# Breast cancer incidence and the air pollution level in the communes of Chile: an ecological study

**DOI:** 10.3332/ecancer.2021.1191

**Published:** 2021-02-25

**Authors:** Jorge Sapunar Zenteno, Pedro Ferrer Rosende, Badir Chahuán Manzur, Isabel Saffie Vega

**Affiliations:** 1 Epidemiology Research Unit, Research Department, Instituto Oncológico Fundación Arturo López Pérez, Cano y Aponte 927, Providencia, Santiago 7500000, Chile; 2 EPICYN Center, Department of Internal Medicine, Facultad de Medicina, Universidad de La Frontera, Temuco 4780000, Chile; 3 Breast Oncology Surgery Unit, Instituto Oncológico Fundación Arturo López Pérez, Santiago 7500000, Chile

**Keywords:** breast cancer, epidemiology, air environmental pollutants

## Abstract

There is evidence linking air pollutants associated with vehicular traffic such as polycyclic aromatic hydrocarbons with breast carcinogenesis. Epidemiological studies have shown conflicting results regarding air pollution and breast cancer risk, which could be explained by the multitude of other risk factors that could affect the association. In Chile, air pollution has reached alarming levels, either due to motorised vehicle traffic or the combustion of wood for heating; therefore, our objective was to evaluate the association between the incidence of breast cancer and the concentration of the main air pollutants monitored in the country.

We carried out a cross-sectional ecological study that evaluated the association between the average incidence of breast cancer in years (2016 to 2018) and the average annual concentration of six atmospheric pollutants in the 5 years prior to the estimation of the rate in communes of Chile, using the population of beneficiaries of Instituto Oncológico Fundación Arturo López Pérez. The annual incidence of breast cancer was 72.21 cases per 100,000 women and it varied significantly in the communes studied compared to the human development index (HDI) and to the proportion of women in the age group at highest risk. Assessing the relationship between the incidence of breast cancer and the average concentration of atmospheric pollutants, we only found a direct correlation between the level of nitrogen dioxide and the rate (*R* = 0.82; *p* = 0.044), whose significance tends to be lost when age and the communal HDI are included in a regression model.

## Introduction

Breast cancer is the leading cause of mortality attributed to malignant neoplasms in female population of Chile (15.59 deaths per 100,000 women per year) and it is also the most common cancer diagnosed in women in developed countries [1, 2]. The World Health Organization estimates that its incidence and mortality will move higher, forecasting 3 million new cases of breast cancer worldwide in 2030 and 900,000 deaths from this cause [3]. It is essential to recognise the factors associated with the risk and prognosis of this disease for its primary and secondary prevention.

Breast cancer is a heterogeneous disease and therefore the associated risk factors vary according to the expression of oestrogen and progesterone receptors in the tumour, the hormonal status of the patient and the invasive nature or in situ of the disease [4, 5]. Among the risk factors for breast cancer, there are modifiable ones such as the use of contraceptives and hormone replacement therapy, nutritional status and a high-fat diet, alcohol and tobacco consumption, exposure to ionising radiation in the chest and reproductive history. Other factors are not modifiable such as age, sex, age at menopause, family and personal history of breast cancer [5]. They can also be classified as major and minor factors according to whether they increase the risk of the disease more or less than 2 times. Among the major risk factors, pathogenic mutations carriage in the BRCA 1 and BRCA 2 genes stood out, having first- and second-degree relatives with bilateral breast cancer, ovarian and breast cancer, breast cancer diagnosed before the age of 50 despite not having demonstrated pathogenic mutations, the presence of a male relative with breast cancer as well as chest irradiation prior to age 30 due to other oncological diseases. Minor risk factors are age, the presence of relatives with a diagnosis of breast cancer after 60 years, nutritional disorders due to excess alcohol intake and exposure to hormonal therapies, among others [6].

In oncology, environmental pollution and in particular atmospheric pollution, which is a known risk factor for some types of cancer such as lung cancer, has gained increasing interest. Polluted air contains polycyclic aromatic hydrocarbons (PAHs) and metals that can act as endocrine disruptors or carcinogens. PAHs have an oestrogenic effect and induce mammary tumours in animal models [7, 8]. In humans, an association has been demonstrated between exposure to respirable particulate matter 2.5 (PM 2.5) and ozone (O3) with higher mammary density, also suggesting an oestrogenic effect on air pollution [9, 10]. Some studies that have investigated the association between air pollution and the risk of incident breast cancer or mortality have had contradictory results [11–15], in part due to their design, the type of pollutant studied and the large number of other factors risk of breast cancer involved.

Chile has a network of air quality monitoring stations located in the main cities or in places where productive activities such as mining, heavy industry and thermoelectric power generation generate pollution. There are cities in which air pollution has reached alarming levels, either due to motorised vehicle traffic or the burning of wood for heating [16].

Our objective was to evaluate whether there is an association between the incidence of breast cancer and the concentration of the main air pollutants monitored in Chile through an ecological study.

## Subjects and methods

An ecological cross-sectional study that evaluated the association between average incidence of breast cancer in 3 years (2016, 2017, 2018) and the average annual concentration of the 5 years prior to estimated incidence of six air pollutants in communes of Chile.

In order to obtain the study population, a health centre in Chile focused on cancer management and that has a captive population was selected. The Instituto Oncológico Fundación Arturo López Pérez (FALP) meets these characteristics. The population cared for in this institution has the possibility of contracting an agreement for coverage of diagnostic benefits and treatment against neoplastic events, called an oncological agreement.

With the approval of the Scientific Ethics Committee of the FALP, the distribution by age, sex and commune of the beneficiaries of the oncology agreement was obtained, as well as the new cases of breast cancer treated at the Institute in 2016, 2017 and 2018. The Incidence estimation was performed for female sex and only breast cancer cases in beneficiaries of the oncology agreement were considered.

To correct the potential inherent bias projecting the incidence of breast cancer from the population in the FALP oncology agreement to the Chilean population, this rate was adjusted by age distribution from 2012 census using the direct method.

The average annual concentration of atmospheric pollutants was based on data records obtained from the air quality monitoring stations distributed in Chile. The pollutants studied were respirable PM 2.5, PM 10, sulphur dioxide (SO_2_), nitrogen monoxide (NO), nitrogen dioxide (NO_2_) and ozone (0_3_). The techniques to determine the concentration of atmospheric pollutants are available on the website of the National Air Quality Information System (SINCA) [16]. The average of the last 5 years prior to the incidence estimate was considered due to the lack of validated data in the oldest records.

Considering that the literature recognises an association between the risk of breast cancer and pollutants linked to motor vehicle traffic [13, 14], we obtained the index of motorised vehicles per capita by commune [17]. By the association between cancer incidence and human development index (HDI), this indicator was obtained by commune [18].

To evaluate the association between the incidence of breast cancer and the average concentration of air pollutants, we selected those communes with a female population of beneficiaries in an oncology agreement greater than 5,000 people and that will have active monitoring stations and validated records.

Statistical analysis was performed with the R program version 3.6.0 (R Core Team, 2018. Vienna, Austria). The correlation between the incidence of cancer with the average concentration of contaminants, the HDI and the proportion of beneficiaries of the oncology agreement in the age group at risk for breast cancer was analysed using Pearson’s correlation coefficient. A *p*-value <0.05 was considered statistically significant. Finally, using multiple linear regression, we evaluated the effect of air pollution on the incidence of breast cancer as a dependent variable, considering age and HDI by commune.

## Results

During the 2016–2018 period, 661 new cases of breast cancer were treated in women with an oncology agreement. The female population in the agreement was 275,768, 310,162 and 339,096 in 2016, 2017 and 2018, respectively. The incidence of breast cancer per 100,000 women in oncology agreement per year was 80.9, 70.3 and 64.9 in 2016, 2017 and 2018, respectively (Table 1).

**Figure 1.**
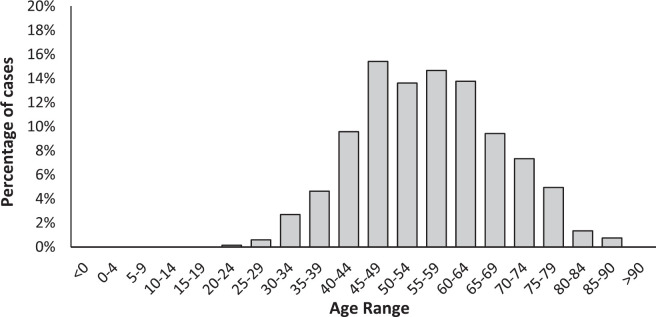
Distribution by age group of breast cancer cases in women beneficiaries of the FALP oncology agreement between 2016 and 2018.

Figure 1 shows the age distribution of breast cancer cases, concentrating most of these between 45 and 65 years (57.5%).

**Figure 2.**
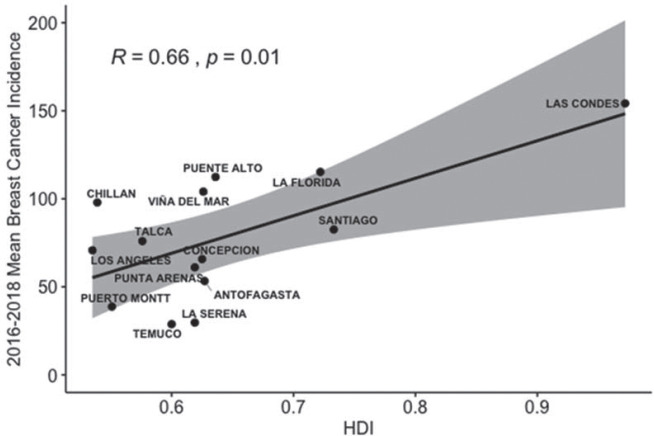
Relationship between the HDI in 2017 and the average incidence of breast cancer per 100,000 people between 2016 and 2018 by commune and age adjusted risk stratum.

By comparing the adjusted incidence of breast cancer among the 14 communes with more than 5,000 beneficiaries of the FALP oncology agreement, Las Condes, Puente Alto, La Florida in the Metropolitan Region and Viña del Mar in Valparaíso region stand out, with rates greater than 100 cases per 100,000. The lowest rates are Temuco in the Araucanía Region, La Serena in the Coquimbo Region and Puerto Montt in the Los Lagos Region (Table 2). By evaluating the relationship between the adjusted incidence of breast cancer and the communal HDI, we found a direct and significant correlation (R = 0.68 *p* = 0.007) (Figure 2).

**Figure 3.**
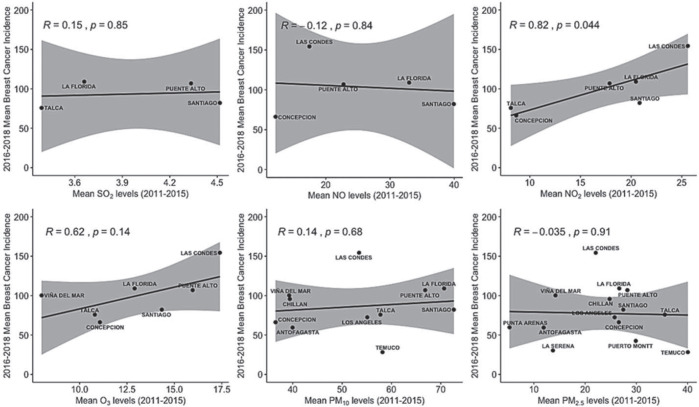
Relationship between average levels of six air pollutants between the years 2011 and 2015 and the average incidence of breast cancer between 2016 and 2018 by commune and age-adjusted risk stratum.

Figure 3 shows the correlation between the average adjusted incidence of breast cancer between the years 2016 and 2018 and the average concentration of atmospheric pollutants between the years 2011 and 2015, ruling out the direct and significant correlation between the NO_2_ level and the rate (*R* = 0.82; *p* = 0.044).

The association with NO2 tends to be lost when analysing the effect of the level of the six air pollutants on the incidence of breast cancer by borough as a dependent variable in a linear regression model that includes the variables age and HDI (Table 3).

## Discussion

According to Global Observatory of Cancer (GLOBOCAN) estimates, breast cancer accounts for 24.2% of new cancer cases in women worldwide. The average incidence of this disease is 54.4 cases per 100,000 women per year in countries with high HDI and 31.3 cases per 100,000 women per year in countries with medium and low HDI. For South America, the GLOBOCAN estimate is 56.8 cases per 100,000 women per year [2]. In Chile, the non-annualised incidence rates of breast cancer available are only projections of the cases registered in three sentinel centres (33.1 and 33.3 cases per 100,000 for the periods 2001–2005 and 2006–2008, respectively, in the Region of Los Ríos, 30.4 cases per 100,000 for the period 2003–2007 in the Province of Bío-Bío and 31.0 cases per 100,000 in the period 1998–2002 in the Region of Antofagasta) [19]. Our study is the first in Chile to obtain a breast cancer incidence rate for a population of women and this was 72.21 cases per 100,000 women per year, a figure in the range observed for countries with a high HDI in the GLOBOCAN projections. The possible demographic differences between the population of women in the FALP oncology agreement and the population of Chilean women were partially corrected by adjusting the rate for the age distribution of the female population from the 2012 census. On the other hand, the oncology agreement is mostly acquired by companies for their workers, which would reduce the risk of selection bias given by individual affiliation.

The 14 communes selected for analysis, considering a population of beneficiaries of the FALP oncology agreement greater than 5,000 people, are distributed throughout the country and have important geographic and socio-economic differences (Table 4). In them, we observe a great variation in the average annualised incidence of breast cancer that ranges from 28.8 cases per 100,000 women per year in Temuco, La Araucanía Region to 154.2 cases per 100,000 women per year in Las Condes, Metropolitan Region.

By evaluating the correlation between the incidence of breast cancer and the average annual concentration of six air pollutants in the previous 5 years, we found an association with the concentration of NO_2_ that however loses statistical significance when using a multiple linear regression model that considers other risk factors. According to SINCA [16] NO_2_ is produced by the burning of fuels at high temperatures that combined with volatile organic compounds in the presence of sunlight forms O_3_ and by doing with water contributes to the production of acid rain and increasing the levels of PM 10 and PM 2.5. The commune of Las Condes exhibits the highest average annual concentrations of NO_2_ and has the highest adjusted average annual incidence of breast cancer. In the case of O_3_, we observed an R coefficient of 0.61 but it did not reach statistical significance. The Sister cohort study in the United States reported an increased risk of ductal carcinoma in situ for high levels of NO_2_ and for invasive carcinoma only in the southern states [14]. Previously, a case–control study conducted in Montreal, Canada, had established an association between NO_2_ contamination and invasive breast cancer [13]. In these studies, NO_2_ was used as a marker of motor vehicle traffic, being the pollutants associated with breast carcinogenesis PAHs and metals [7]. The commune of Temuco, in the Region of La Araucanía, has high levels of contamination by PM 2.5 attributable to the combustion of firewood for heating but a low incidence of breast cancer.

One of the limitations of our study is the lack of validated records of all pollutants in the air quality monitoring stations. In those communes that are part of the largest conurbations in Chile (Santiago, Valparaíso and Concepción), the registry is complete, while in communes of smaller conurbations it is limited to contaminants previously recognised as critical. The direction of this bias would possibly not affect the results since the communes that did not register NO_2_ have low incidences of breast cancer. There is also the possibility of selection bias in the beneficiaries of the oncology agreement, since they could have hired it because they have risk factors for cancer, increasing the incidence of the disease in relation to the general population. However, the rate found is similar to that proposed by GLOBOCAN [2] and as explained in Subjects and Methods section, the oncology agreement is a collective plan for companies. Our study is population-based and unfortunately the characterisation of this population in relation to risk factors for breast cancer is incomplete, being only able to analyzs the effect of age and HDI on the association between levels of atmospheric pollutants and community incidence of the neoplasia. This constitutes the main limitation of the study since it is not possible to know if the frequency of other risk factors is comparable between the communes studied. Although the concentration of contaminants was an average of the 5 years prior to the estimation of the incidence, it cannot be guaranteed that the disease (breast cancer) did not exist prior to exposure, which is a limitation of all studies of cross-section.

## Conclusion

In conclusion, the incidence of breast cancer in the studied population is similar to that of countries with high HDI and there is a direct correlation between the frequency of breast cancer and an air pollutant mainly related to motor vehicle traffic; however, this association is lost by considering the effect of variables such as age and HDI. New studies are required whose design makes it possible to evaluate the effect of other risk factors and establish the temporality of the association between exposure to air pollutants and incidence of breast cancer.

## Conflicts of interest

None.

## Funding statement

None.

## Figures and Tables

**Table 1.** Beneficiary population of the FALP oncology agreement in the years 2016, 2017 and 2018, average age and direct age-adjusted incidence according to projections of the 2012 census.

	2016	2017	2018
Female population oncological agreement	275,768	310,162	339,096
Cases	223	218	220
Average age of cases (range)^a^	55.8 (24–87)	54.6 (27–88)	56.8 (26–88)
Incidence by per 100,000 (CI_95%_)	80.9 (70.6, 92.2)	70.3 (61.3, 80.3)	64.9 (56.6, 74.0)
Age-adjusted incidence (CI_95%_)^b^	82.6 (69.5, 97.5)	70.2 (59.6, 82.1)	71.2 (59.8, 84.1)

a (*F*_(2,662)_ = 1.86; *p* = 0.157)

b According to census projections 2012

**Table 2.** Beneficiary population of the FALP oncology agreement by commune and breast cancer incidence for the years 2016, 2017 and 2018, age adjusted risk group.

Commune	Region	Adjusted incidence 2016	Adjusted incidence 2017	Adjusted incidence 2018	Adjusted incidence per 100,000 2016–2018
Antofagasta	Antofagasta	49.3 (15.9, 115.5)	73.2 (31.6, 144.2)	55.9 (22.4, 115.3)	59.5
Chillán	Ñuble	132.4 (57.1, 261.3)	73.8 (23.9, 172.2)	80.8 (29.6, 175.9)	95.7
Concepción	Biobio	106.5 (39.1, 231.8)	48.7 (10, 142.4)	43.4 (8.9, 127)	66.2
La Florida	Metropolitana	97.9 (41.9, 194.2)	124.3 (61.5, 223.7)	105 (50, 193.9)	109.1
La Serena	Coquimbo	20.4 (0.5, 113.9)	55.4 (11.4, 161.9)	15.4 (0.4, 85.9)	30.4
Las Condes	Metropolitana	114.1 (54.7, 209.8)	181 (105.4, 289.8)	168 (96, 272.8)	154.3
Los Angeles	Biobío	71.5 (19.5, 183)	66.5 (18.1, 170.3)	79.5 (25.8, 185.5)	72.5
Puente Alto	Metropolitana	150.7 (75.1, 269.8)	92.8 (40, 183)	77.1 (30.9, 159.2)	106.9
Puerto Montt	Los Lagos	76.5 (24.8, 178.5)	37.9 (7.8, 111.2)	13.2 (0.3, 73.5)	42.5
Punta Arenas	Magallanes	38.1 (4.6, 137.7)	36.4 (4.4, 131.5)	104.6 (38.4, 227.6)	59.7
Santiago	Metropolitana	85.7 (34.4, 176.5)	98.7 (45.1, 187.4)	62.2 (22.8, 135.3)	82.2
Talca	Maule	62.3 (17, 159.5)	84.6 (31.1, 184.2)	80.6 (29.6, 175.4)	75.8
Temuco	La Araucanía	16.6 (0.4, 92.4)	14.7 (0.4, 81.8)	53.5 (14.6, 137)	28.2
Viña del Mar	Valparaíso	77.6 (21.1, 198.9)	98.3 (36, 214.2)	124.9 (53.8, 246.5)	100.3

**Table 3.** Linear regression analysis for the pollutants effect on the breast cancer incidence.

	Not adjusted value		Age adjusted value		Adjusted value by population at risk		Adjusted value by HDI	
Pollutant	*β* (SE)	*p*-value	*β* (SE)	*p*-value	*β* (SE)	*p*-value	*β* (SE)	*p*-value
SO_2_	4.94 (26.35)	0.869	5.14 (37.2)	0.912	11.76 (12.1)	0.509	−1.77 (41.1)	0.973
NO	−0.27 (1.72)	0.885	−0.08 (1.78)	0.968	−0.24 (2.16)	0.921	−0.16 (1.28)	0.912
NO_2_	3.85 (1.29)	**0.041**	3.45 (1.90)	0.167	3.85 (1.50)	0.082	2.13 (2.51)	0.460
O_3_	5.51 (3.30)	0.156	4.18 (3.20)	0.262	5.98 (3.64)	0.176	0.681 (3.95)	0.871
PM_10_	0.46 (0.85)	0.600	0.30 (0.66)	0.664	0.12 (0.87)	0.892	−0.02 (0.71)	0.976
PM_2.5_	−0.09 (1.09)	0.938	−0.26 (0.77)	0.742	−0.40 (0.97)	0.688	0.17 (0.86)	0.846

SE: standard error; PM: respirable particulate matter

**Table 4.** Geographic and demographic characteristics of the 14 communes of Chile included in the study.

Commune	Region	Latitude (SL)	Altitude (m ASL)	Population	HDI
Antofagasta	Antofagasta	23°38′	40	425.725	0.627
Chillán	Ñuble	36°36′	124	184.739	0.539
Concepción	Bíobío	36°38′	12	223.574	0.625
La Florida	Metropolitana	33°33′	784	366.916	0.722
La Serena	Coquimbo	29°54′	28	249.656	0.619
Las Condes	Metropolitana	33°25′	709	294.838	0.972
Los Ángeles	Biobío	37°28′	139	202.331	0.535
Puente Alto	Metropolitana	33°37′	698	625.501	0.636
Puerto Montt	Los Lagos	41°28′	14	245.902	0.551
Punta Arenas	Magallanes	53°28′	10	131.592	0.619
Santiago	Metropolitana	33°27′	579	404.495	0.733
Talca	Maule	35°25′	102	220.357	0.576
Temuco	La Araucanía	38°44′	122	282.415	0.600
Viña del Mar	Valparaíso	33°01′	2	334.248	0.726

SL: south latitude; mASL: meters above sea level
